# Understanding how community-dwelling persons with early dementia perceive health and community services: Informing the dementia strategy of Newfoundland and Labrador, Canada

**DOI:** 10.1177/14713012241284693

**Published:** 2024-09-13

**Authors:** Karen Parsons, Joanne Smith-Young, April Pike

**Affiliations:** Faculty of Nursing, 7512 Memorial University, Newfoundland and Labrador, Canada

**Keywords:** dementia, health services, older adults, qualitative research, support services

## Abstract

**Background:**

With an increased aging population, the number of individuals with dementia is expected to rise. The onset of dementia marks the start of negotiating access to a wide range of health and social services to find practical and emotional supports to deal with the management of changes and subsequent challenges that individual with dementia face. The toll of dementia goes beyond the health care system, affecting families and caregivers’ quality of life. This places more pressure on family caregivers and health care institutions to provide services for affected individuals. It is important to understand the service needs of this population to enable them to live at home longer, contribute to society and maintain a positive quality of life.

**Aim:**

To increase understanding of how persons living at home with early dementia and their caregivers/significant others currently perceive and interact with health and community-based services and service providers.

**Methods:**

A qualitative descriptive approach was used to explore the experiences of individuals with early dementia and care providers with health and community-based services using semi-structured interviews and content analysis.

**Results:**

Participants included 16 individuals 50 years and older with mild/early dementia living at home, 22 informal caregivers of individuals with mild/early dementia, and 5 key community informants (community health nurses and social workers). Four thematic categories of barriers and two thematic categories of facilitators for access to and uptake of supportive services were identified. Five strategies to inform the development of an action plan to enhance access to, and uptake of, supportive services were determined.

**Conclusions:**

Early recognition of dementia through education and publicity enhanced public awareness, attention, and social inclusion with dementia-friendly neighborhoods and facilities need to be considered to achieve effective dementia-related services. Inclusion and recognition of the wishes of persons with dementia is key.

## Background

According to the Alzheimer’s Society of Canada, as of January 2024, there were as estimated 733,000 people living in Canada with dementia; this number is expected to increase by 187% by 2050. More than 60% of these individuals are living at home (CIHI, 2024), and of those 80% have mild to moderate impairment and wish to live at home for as long as possible (CIHI, 2024). In the Canadian province of Newfoundland and Labrador (NL) there are approximately 10,000 people living with dementia (Government of NL, 2023). NL has the fastest aging population in Canada, and the number of individuals with dementia is expected to increase to 14,486 by 2035 (Government of NL, 2023).

Early symptoms of dementia include changes in memory, communication, judgement, abstract thinking, and mood (Alzheimer’s Society of Canada, 2024). These changes not only mark a time of seeking diagnosis and medical management but are also mark the start of negotiating access to a wide range of health and social services. Timely access to supportive services is necessary to enable improvements in dementia care (Gaugler et al., 2005; Jelley et al., 2021), increase QOL, reduce informal caregiver burden, and reduce health care costs (Phelan et al., 2012; Weimer & Sager, 2009). However, according to the findings of a systematic review by Low et al. (2018) on the subjective experience of people with early dementia, the need to find practical and emotional supports to deal with the many changes and subsequent challenges that dementia incurs is often fraught with disillusionment and disempowerment. People with dementia are often not told their diagnosis, are often provided insufficient information, and treatments are often limited to medications with little support for managing memory, other symptoms, and other forms of post-diagnosis support. Further, often lacking in the community are a range of higher-level services for social needs (Jelley et al., 2021).

With appropriate supports, older adults with early-stage dementia can live at home longer, contribute to society and maintain a positive QOL (Tam-Tham et al., 2016). Older adults with dementia indicate that support services are crucial to maintain activities of daily life (Forbes et al., 2006). The support of caregivers is crucial. Older adults with dementia who do not have a caregiver at home or who have a caregiver that is unable to provide care are twice as likely to enter long-term care (CIHI, 2024). There has been a plethora of research in the past 30 years on dementia and caregiver burden. However, research on the needs of both persons in the very early stage of dementia and their informal caregivers is limited.

In the province of NL Canada, the Department of Health and Community Services is responsible for the overall strategic direction and priorities for health and community services and includes community-based service for older adults, including those with dementia, such as the provincial home support program that supplements services provided by family and friends (Government of NL, n.d.). These services include the home support program that aims to fulfill the daily necessities of this population. These services can be purchased privately, or may be subsidized from public funds, to a maximum financial ceiling. Referral for services can be initiated by anyone including the individual requiring service. To be eligible for financial subsidy the individual must undergo a functional and financial assessment.

In 2019, the Canadian Federal Government released a dementia strategy (Government of Canada, 2019). Provinces and territories collaborated with the Federal Government to inform development of the strategy, and actively engage in supporting its implementation. One of the primary objectives of the strategy was to improve the quality of life for people living with dementia and their caregivers. To that end, the Alzheimer Society of NL collaborated with the government of NL to develop the Dementia Care Action Plan 2023–2026 (Government of Newfoundland and Labrador, 2023). An important initiative of the Action Plan was to ensure that people with dementia and their caregivers are included, engaged, and informed of all aspects of the partnership. The government of NL launched a comprehensive public engagement process so that the best practices in dementia care would reflect a person-centered and strengths-based approach and build on existing strategies such as the Health Accord NL (Health Accord NL, 2022). An advisory council was subsequently employed to lead the Dementia Action Plan; members included patients, key community members, policy makers, and the Alzheimer’s Society of NL. The importance of including caregivers of persons with dementia as community stakeholders for improving care and services is not new (Liebzeit et al., 2023). However, there has been an increasing call for people with dementia themselves to be included in research (Gove et al., 2018).

The Dementia Action Plan consists of four foci. One of four was to improve the supports and services that people need to live well with dementia. During the onset of dementia, individuals living with dementia and their families/significant others often do not realize that they and/or their family member are candidates for community supportive services (Dixon-Woods et al., 2006). Further, they are often unaware of available services, have problems finding relevant information and/or are unable to access these services (Ducharme et al., 2014; Millenaar et al., 2016; Parsons et al., 2013) or perceiving them as not yet necessary (Jelley et al., 2021). Prompted by Canada’s National Dementia Strategy and simultaneous to the development of the Dementia Care Action Plan by the NL government, we launched our independent research investigating the supports and services that people need to live well with dementia; specifically people with early-stage dementia. The findings of our research later brought us to the table of the advisory council with research-based evidence to inform the development of sustainable actions to enhance access to, and uptake of, supportive services for older adults and their families/significant others living at home with dementia in NL. We plan to publish the results of this in a subsequent paper.

## Research objectives

The primary purpose of our study was to increase understanding of how persons living at home with early-stage dementia, their caregivers/significant others and key informants such as nurses and social workers currently perceive access to and uptake of health and community-based services in NL. Our ultimate goal was to help inform the Dementia Care Action Plan on one of its foci, to improve supports and services that people need to live well with dementia.

The research objectives were:

1. To identify barriers and facilitators related to access and uptake of formal supportive services that are necessary to improve and sustain health and QOL for older adults with mild/early dementia living at home.
2. To identify best strategies to inform the development, implementation, and sustainability of an action plan to enhance access to and uptake of supportive services to improve health and QOL.

## Methods

### Study design

We used a qualitative descriptive method (Sandelowski, 2000) to explore the experiences of individuals with early-stage dementia and care providers with health and community-based services. Qualitative description is an appropriate methodology to conduct research when comprehensive descriptions of phenomenon are desired that are grounded in the participants’ voices (Bradshaw et al., 2017; Sandelowski & Barroso, 2007). By staying true to participants’ own words, qualitative description enables researchers to report findings in a clear and logical way, in everyday language (Sandelowski, 2010). To that end, qualitative description uses a purposive sample, semi-structured interviews and content analysis (Kim et al., 2017). Qualitative research is essential to evidence-based practice and contributes to identifying promising implementation strategies (Sandelowski et al., 2006).

### Ethics

This study was approved by the Health Research Ethics Authority of Newfoundland and Labrador [Health Research Ethics Board (HREB) reference number 2019.173]. Ethical considerations included describing any risks and inconveniences in participating in the study and freedom to withdraw from the study at any time without repercussions. Personal identities were anonymized during transcription. All applicants provided informed consent. In Canada, adults are presumed to be capable of consent unless there is reasonable grounds to presume otherwise. The law recognizes the right of every person to self-determination. However, only older adult participants who appeared able to understand the nature of the research and could dialogue with the researcher were consented and included. As part of the consent process the study was explained at two points in time; first, during the initial contact via phone or email and second, prior to the start of the interview. At both times the purpose of the research was explained and participants were given the opportunity to ask any questions. They were informed that they could make the decision to refuse to answer any interview questions or stop the interview at any time.

Participants were told that the personal health information or personal information collected about them would have directly identifiable information removed (i.e., name, address) and replaced with a code or with a “study number”. There was a master list linking the code numbers to names. The researcher is responsible for keeping it separate from the personal information. Study information collected during the study would kept at this site and stored in a secure, locked place that only the study researchers could to access.

Interviews were initially intended to be conducted in person, but because of COVID-19 data collection was halted after only seven in-person interviews. We received approval for an amendment from HREB to conduct the interviews either by telephone or virtually and we resumed data collection at that time. Consent for the first in-person interviews was obtained prior to the start of the interviews and participants were provided a copy of the consent form. For interviews conducted by telephone or virtually, consent was obtained virtually and participants later received a copy of the consent via mail. The Principal Investigator and Research Assistant were sufficiently experienced in the field of dementia and aging to determine if the older adults were able to understand the study and participate. No one was excluded based on cognitive decline. This was attributed to the participants having only mild cognitive impairment.

During data collection it can be challenging to keep older adult participants with early dementia on track, therefore, it was important that we confirmed information and repeated questions. Furthermore, older adults may tire easily therefore, periodic breaks were offered to accommodate the highest comfort level. We informed participants that they could stop the interview or leave the study at any time.

### Participants and recruitment

The research took place in five communities (one urban and four rural) in Eastern NL, Canada. Inclusion criteria for individuals with dementia included those who (a) self-identify as experiencing symptoms of early-stage dementia, with or without having received a cognitive-related diagnosis and (b) live at home. It is believed that a lack of a formal diagnosis of dementia would not influence older adults’ perception of need for services. The presuppositions of the research team, derived from previous experience with individuals with early-stage dementia, is that such individuals who respond to the call to be a participant in a study of early-stage dementia believed they fell into this category of dementia. The perceptions of the participants regarding their own cognitive abilities in everyday functioning has been shown to be sensible and reliable for early dementia detection (Carr et al., 2000). Given the increase in dementia literacy in the general population, which has expanded to include not only knowledge about dementia, but also the individual’s capacity to translate knowledge into values, beliefs, and actions. Further, individual, are now more than every empowered to influence the prevention, detection and care trajectory of dementia (Nguyen et al., 2022).

Inclusion criteria for informal caregivers included individuals who identified as the family member or significant other in the role of primary caregiver for individuals experiencing the symptoms of early-stage dementia or diagnosed with early-stage dementia.

Key informants are those professionals employed by the local health authorities that are governed by the Department of Health and Community Services, responsible for the overall strategic direction and priorities for health and community services that includes community-based service for seniors, including those with dementia. These services include the provincial home support program that supplements services provided by family and friends. Inclusion criteria for key informants included healthcare professionals such as registered nurses and social workers who work with, and were knowledgeable about, individuals experiencing the symptoms of early-stage dementia or who have been diagnosed with early-stage dementia living at home who have sought formal supportive services.

We used diverse methods of purposive sampling. Recruitment of older adults and caregivers included posting flyers in seniors’ community settings, the Alzheimer’s Society of NL website, and public locations such as grocery stores, department of community services, and doctors’ clinics. Recruitment for key informants consisted of an e-mail to key community organization such as the Alzheimer’s Society of NL, the Seniors’ Resource Centre, the College of Registered Nurses of NL (CRNNL) and the NL Association of Social Workers, requesting distribution on their list serves. We also conducted a public service announcement on local radio stations. Recruitment of key informants, especially of nurses was challenging as data collection occurred during the COVID19 pandemic and many community health nurses were severely stretched in their duties with little extra time for participation in research. Participants who were interested in taking part in the study contacted the research assistant who arranged for an interview.

Participants included individuals 50 years and older with mild**/**early-stage dementia living at home (*n* = 16), informal caregivers of individuals with mild**/**early dementia (*n* = 16 and 22, respectfully) [see Table 1], and key community informants, community health nurses and social workers (*n* = 5). [See Table 2.] There was no link between participants with dementia and the caregivers except for caregivers 5a and 5b who were a married couple who wished to be interviewed together.

**Table 1.** Individuals with early-stage dementia and caregivers.

Participant ID	Gender	Age	Age at First Notice of Memory Loss	Marital Status	Employment Status	Total Household Income	Age of Family Member with ED	Relation-ship to Family Member with ED
Individuals With Early-Stage Dementia								
1	F	59	59	Married/Common Law	Retired	$48,000-$58,000		
2	F	69	57	Married/Common Law	Retired	$15,000-$25,000		
3	F	74	73	Divorced/Separated/Widowed	Retired	$37,000-$47,000		
4	M	75	73	Divorced/Separated/Widowed	Retired	$37,000-$47,000		
5	M	77	76	Married/Common Law	Part-time	$26,000-$36,000		
6	M	84	83	Married/Common Law	Retired	$48,000-$58,000		
7	F	75	75	Married/Common Law	Retired	$26,000-$36,000		
8	F	61	60	Married/Common Law	Retired	Over $80,000		
9	F	75	73	Divorced/Separated/Widowed	Retired	$59,000-$69,000		
10	M	83	74	Divorced/Separated/Widowed	Retired	$48,000-$58,000		
11	M	54	47	Married/Common Law	Full-time	Over $80,000		
12	F	59	57	Divorced/Separated/Widowed	Full-time	$26,000-$36,000		
13	F	60	59	Married/Common Law	Retired	$48,000-$58,000		
14	F	63	47	Married/Common Law	Retired	$37,000-$47,000		
15	M	60	50	Married/Common Law	Retired	$59,000-$69,000		
16	M	70	68	Married/Common Law	Retired	Over $80,000		
Caregivers								
1	M	61	58	Married/Common Law	Retired	$37,000-$47,000	60	Husband
2	F	55	72	Married/Common Law	Full-time	Not identified	80	Daughter
3	F	48	63	Single	Full-time	Over $80,000	66	Daughter
4	F	55	82	Married/Common Law	Full-time	Over $80,000	84	Daughter
5a	F	55	72	Married/Common Law	Full-time	$48,000-$58,000	74	Daughter
5b	F	51	72	Married/Common Law	Full-time	$37,000-$47,000	74	Daughter
6	F	68	86	Married/Common Law	Retired	$70,000-$80,000	94	Daughter
7	F	41	70	Married/Common Law	Full-time	Over $80,000	76	Daughter-in-law
8	F	75	67	Married/Common Law	Retired	$48,000-$58,000	70	Wife
9	F	57	82	Married/Common Law	Un-employed	$48,000-$58,000	87	Daughter
10	F	75	67	Married/Common Law	Retired	$48,000-$58,000	75	Wife
11	F	49	82	Single	Full-time	Over $80,000	De-ceased	Daughter
12	F	55	48	Single	Retired	Over $80,000	56	Sister
13	F	67	66	Divorced/Separated/Widowed	Retired	$37,000-$47,000	71	Sister
14	F	70	79	Married/Common Law	Retired	Over $80,000	83	Wife
15	F	63	68	Married/Common Law	Retired	$37,000-$47,000	71	Wife
16	M	74	69	Married/Common Law	Retired	$48,000-$58,000	72	Husband
17	F	57	73	Married/Common Law	Retired	Over $80,000	De-ceased	Daughter
18	F	51	75	Married/Common Law	Full-time	Over $80,000	81	Daughter
19	F	74	64	Married/Common Law	Retired	$48,000-$58,000	76	Wife
20	F	70	70	Married/Common Law	Retired	$26,000-$36,000	73	Wife
21	F	59	89	Divorced/Separated/Widowed	Full-time	$26,000-$36,000	91	Daughter
22	F	60	83	Married/Common Law	Retired	$48,000-$58,000	93	Daughter

**Table 2.** Key informants.

Participant ID	Gender	Professional Role	Length of Time in Present Position	Setting
1	F	Community Health Nurse	23 years	Rural
2	F	Intake Social Worker	1 year in present position/22 years in Community Support Program	Urban and Rural
3	F	Social Worker	8 years	Rural
4	F	Coordinator	4 years	Urban and Rural
5	F	Social Worker	23 years	Rural

### Data collection

Data collection took place from February, 2020 to July, 2022. We designed three separate semi-structured interview guides for the interviews; one for the older adults, one for the caregivers, and one for the key informants. The interview guides consisted of 15 open-ended questions with prompts. Questions included what services were sought due to dementia, facilitators and barriers to services, and potential strategies that might facilitate knowledge of and access to services. The interviews were conducted by the lead author and/or the Research Assistant (RA). Interviews were audio-recorded with consent of the participants.

### Data analysis

The audio-recorded interviews were digitally transcribed verbatim and read several times by two members of our research team. Original transcripts were imported into NVIVO for storage and coding. After much immersion in the data, the interviews were analyzed using content analysis. The initial coding strategy involved categorizing data according to the relevant research question. Data were reduced to coded units were and then condensed and reflected upon and were then clustered into categories according to shared characteristics (Milne & Oberle, 2005). Critical review of coding is an on-going requirement. Because data collection and analysis occurred simultaneously, new codes continued to emerge that required reexamination of existing ones. The categories did not define the data. Rather, data define the categories that are only as descriptive, as the data itself. The categories were contextualized using anonymous illustrations provided by participants. Disagreements between researchers in coding were rare and when they did occur they were discussed until consensus was reached. All data were subsequently brought together for final analysis.

### Rigor

Strategies to increase rigor were those proposed by Whittemore et al. (2001) and Thompson Burdine et al. (2020). First, researchers with a strong qualitative research background and experience conducted all interviews. We ensured authenticity, or attention to the voices of participants. We paid attention to any conscious and unconscious bias of the research team members during all phases of the research process by keeping memos/notes/journals and maintaining dialogue with team members. Second, we ensured credibility of how believable the results were. For example, one strategy used was to have more than one group of participants (i.e., older adults with dementia, family caregivers, key informants) so that the depth and breadth of participants’ experiences were captured. By interviewing different groups of participants we were able to more fully understand, clarify and refine the experience of how access to and uptake of health and community-based services in NL is experienced. Third, we ensured criticality, the critical appraisal of every decision made throughout the research process was achieved by having more than one researcher involved in all phases of the research, and fourth, integrity was demonstrated by ongoing reflection and self-criticality of the researchers. Finally, all members of the research team independently scrutinized the transcripts and compiled an audit trail of the analytical process.

## Findings

Analysis of the data revealed four thematic categories of barriers and two thematic categories of facilitators for access to and uptake of supportive services. We also identified five strategies to inform the development of an action plan to enhance access to, and uptake of, supportive services.

### Barriers

Barriers to services were generally of two types; limited knowledge of dementia and services available, and issues perceived or encountered within the healthcare system, which includes health and community services, and social services. Barriers were, I don’t know; I don’t have Alzheimer’s disease; not that bad; and the healthcare system.

#### I don’t know

A common barrier identified by many of the older adults and caregivers who were beginning their dementia journey was a lack of knowledge. Not knowing about what services were available, or if there were even services available for early dementia, and/or how to access services, and what services they might need in the future, was a common theme.

Like I didn’t know I could have anybody help me, I didn’t. I had people saying oh, you can get hours of help. You can get this and you can get that. I’m like, ‘Okay, tell me what I got to do’. You don’t know…It’s like I’m looking for something and I don’t even know and that’s what I say. (Caregiver 6)

Well, I am thinking that I will need services in the future but I’m not sure what services. Will I need services of a social worker? So down the road I don’t know what’s going to happen and I feel insecure because I don’t have anything in place but I don’t know what I’m supposed to have in place. (Caregiver 10)

#### I don’t have Alzheimer’s disease

Six of the participants interviewed believed that a diagnosis of dementia did not equate to having Alzheimer’s disease. While this is true, it led to a misunderstanding around the services provided by the Alzheimer’s Society. Further, there seemed to be a stigma associated with *Alzheimer’s disease* that was not necessarily associated with *dementia* and this proved to be a barrier for many in reaching out for help. One participant mentioned that the Alzheimer’s Society was for individuals who were much further along in their dementia journey and not for someone like her who was experiencing minimal forgetfulness.

I understand what they [Alzheimer’s Society] do. I’ve seen them on the news. I’ve seen them in advertisements, supported their tea, that sort of thing, so yes, I certainly do know what they do. I guess when I think of them I think of them in terms of people who are much further along the road than I am and perhaps not, perhaps they deal with people who are just sort of taking a tentative first step. (Early dementia participant 3)

When asked why she felt this way she stated that the Alzheimer’s Society was for people with Alzheimer’s disease, which she did not have. She also noted that given the broad array of services provided the name of the organization should be reconsidered.

There are other kinds of dementia other than Alzheimer’s and if I wait until I’m diagnosed with Alzheimer’s that’s not now and it may never be. Alzheimer’s is a form of dementia as far as I understand. So yes it certainly would I think contribute to people thinking that okay, if I get diagnosed with Alzheimer’s I go to see these people and they’ll give me some help but I would not have thought of it to go at the very first step of memory loss. (Early dementia participant 3)

#### Not that bad

Early dementia was viewed as different, not fitting within the services that were available for more advanced dementia. Many of the older adults and their caregivers believed that services that were available were for those with more advanced dementia and not available to individuals like themselves in early stages of the disease.

I don’t know. I guess I never thought that at the point I’m at anyway that there’s much in the way of any help available other than diagnosing whether you actually have early onset of something and if you do, what treatment is available? I’ve never even considered that there might be community support for someone like me. (Early dementia participant 12)

One caregiver felt that the biggest barrier to considering accessing services was the fact that her mother, who was in the early stage of dementia, was relatively healthy and only 63 years old. Yet, her mother was often forgetting important instrumental everyday activities, such as taking her medications and performing good self-care. This left the daughter wondering if care was necessary.

Mom is 63 years old she’s a relatively healthy woman. I mean she can get up and make her bed and cook, well I suppose what is defined as a meal. She was able to feed herself and keep her apartment clean and stuff like that. I mean she’s really technically not that bad, and doesn’t need someone to assist her in that way. But like I said, it was more of the mental part of it. And like I said, we could never confirm if she did take her medication or if she was actually in fact eating or self-care, if she was showering… And I said to have someone in there for homecare is kind of pointless, so you’re going to have someone sitting around going okay, you take your medication. It was kind of pointless. (Caregiver 3)

Resistance to care was seen to be related to believing they did not require formal or informal services, wanting to maintain their independence and/or privacy or prideful not wanting to admit they needed help.

I have talked to dad about that but mom is so resistant to having anyone. She doesn’t even want my sister in doing light tasks for her so she’s certainly not going to be receptive to some stranger coming in and helping even though I think it would be the best thing for both of them. It would ease some of the strain. (Caregiver 4)

She [wife] recognizes it [memory problems] but doesn’t do anything about it. [Because of] Pride… No, he won’t go to the doctor. No one brings him. If he says he doesn’t want to go, he doesn’t go. He hasn’t even been officially diagnosed. Therefore, no supports have been put in place because no one is acknowledging that he has issues, that he has problems. (Caregiver 7)

#### The healthcare system (department of health and community services)

The healthcare system was in itself seen as a barrier to services for early-stage dementia. Barriers in the healthcare system encompassed broad systemic barriers such as lack of services, difficulty navigating the system and high cost. Another barrier identified was a that health care providers were sometimes unconcerned or unwilling to acknowledge or address the problem. Many participants, especially those living in rural areas noted the lack of available services.

I think there are only 550 people living here now in this community. She [Nurse Practitioner] comes two days a week, Monday and Tuesday. We don’t have anything in regards to services, my dear. The next closest one is in [larger community]. That’s an hour away or probably a bit more. They don’t have services for dementia. (Caregiver 20)

A lack of services for those experiencing dementia was verified by key informants.

Yeah, well I was talking to, myself and the social worker had a conversation, well we had lots of conversations about this but the numbers are high here and there is very little available for them. We usually get calls for support to help support them either early on or sometimes a little later but even early on, there’s very little here. But with regards to the referrals to the Alzheimer’s Society that type of thing, other than that anyone who usually wants services we’re it. (Key Informant 1)

Several caregivers noted that the healthcare system was not designed for older adults, and certainly not for approaching the problem of dementia.

But yeah, there’s got to be a better way to make this part of aging, I guess because it seems to me more and more people are experiencing it [dementia] and not to be afraid of it. So if we could somehow approach it that way because the first thing people think of is that you’re going to end up in a home because that’s what my dad has talked about…I think it needs to be somehow out there that you don’t have to go to a GP to get in to be seen or to be helped. There’s got to be an easier way to go about this because again, it’s hidden. (Caregiver 2)

Caregivers also noted that while homecare for people with dementia was important and possibly available if needed, it was not an option due to cost.

It’s just a matter of cost that’s the issue…I guess if I did get someone in and they said, ‘Yeah she is someone who could benefit from having home care’ we kind of know that. [laughing]. And then, it just becomes are you going to qualify for the government program to get assistance with it and I don’t think they would from what information I’ve gotten from people I’ve spoken with so, it’s kind of like a moot point (Caregiver 4).

Although homecare was being used by one family for their mother with early-stage dementia, her daughter felt that the number of hours were insufficient to properly meet her mom’s needs.

It’s the meals that are the issue and I mean yes, its 4 ½ hours but you’re only getting help with one meal out of that…So in reality, I think there should be more but they won’t approve more. (Caregiver 5a)

Several participants, although mostly satisfied with their care providers, noted a lack of concern or understanding from their family physician around dementia and/or an unwillingness to confront the problem.

I’ve said a couple of times, ‘Dr. [name], you know I’m losing it’ He will just joke about it and he’ll say, ‘Look, any memory issues you have are like age-related dementia.’ And I said, ‘What?! Dementia?’ And he said, ‘Like you’re 75. You’re not going to recall things as quickly and so on, like that.’ But he has never seen anything that would cause him to feel some sense of alarm or something that was happening. (Early dementia participant 9).

### Facilitators

Two categories of facilitators for supportive services were identified: the healthcare system and the Alzheimer’s Society.

#### Healthcare system

While some aspects of the healthcare system were viewed as a barrier for some participants in our study, it was a positive experience for others and facilitated access to services.

I first made contact with [name], the clinical coordinator, someone in that area and they would say when I would explain to them the situation that mom was in and they would say, you might want to check with this person or that person or they might have some information that can be helpful for you. So they kind of spread their word around altogether. They’re all kind of working together on that so I thought that was good. (Caregiver 4)

#### Alzheimer’s society

While there were accounts of not understanding the role and services provide by the Alzheimer’s Society, the Alzheimer’s Society was regarded by many participants as an important resource for obtaining information and psychological support. Caregivers also noted the ease of accessibility.

I have to say that [Alzheimer’s Society] was really fabulous. It was an excellent program [first-link program]. What I found is there were a lot of things about it that were really excellent. I mean obviously, the information provided was really good. It was wonderful that the speakers were I would say almost 100% of them were actually people who were there for their professional expertise but they also had personal experience. (Caregiver 14)

One caregiver noted how important it was to her that the Alzheimer’s Society had treated her kindly, listened to her concerns, and offered advice in a very intimate manner over a cup of coffee.

We just met over a cup of coffee and I talked to her about my concerns and she encouraged me to make that move and to think about myself and how I would live in a future that didn’t involve [husband]. (Caregiver 14)

### Strategies to address barriers and enhance services

Participants readily suggested five key strategies to address barriers and enhance services. Many of the strategies suggested by the participants in this study were as expected, such as the need to hire more healthcare workers in order to increase services. Other strategies suggested stemmed from the obstacles they faced and the lack of resources they encountered. Strategies suggested were having an easier way of accessing information, having a navigator to assist them with a care pathway, and receiving preferential treatment when seeking medical services. The Alzheimer’s Society was often singled out as a key source of information and support. However, two participants felt that the Alzheimer’s Society could expand their scope to include the atypical younger victim of dementia. Increased visibility around Alzheimer’s disease was also noted as a potential strategy.

#### Hiring more healthcare providers

Key informants including nurses and social workers noted that their caseload had doubled, increasing the demands placed upon them. The need for health authorities to hire more healthcare professionals was a common theme.

When I started first, as a nurse, because we have other programs apart from the home care, when I started first we could have 10 on our caseload, we could have 14, or 20 but now each of us here got at least 40 each. The programming has increased, and the demands of the program have increased. There’s more for us to do with it than we did years ago and then plus all the other nursing programs as well thrown in on top of it is a lot! Plus, I’m getting older too! (Key Informant 1)

#### Ready-to-hand information

Participants stated that one of the most important things they felt they needed was easy access to information. Caregivers stated that getting started and knowing what to do was the most difficult part of the caregiving process.

Well, if there was some place that we could go, if say myself, dad, my brother and my sister, if the three of us could go in and just say, ‘Look, this is what’s happening to mom or to my wife and we don’t know how to approach it and we don’t know how to handle it, what do we do? Can you give us some guidance?’ (Caregiver 2)

Given that navigating the early stages of dementia was often problematic, it is not surprising that the need for a single individual to act as navigator in the process was noted by caregivers as important.

What we would have liked for somebody to say to us, and this somebody should have been from the Alzheimer’s Society in my mind, to come in, your sister has dementia. Now every doctor who is diagnosing should know after the diagnosis I should send the family member over to [name at the Alzheimer’s Society.] And [name’s] team should be able to sit down and say, your sister has dementia. This is everything that is available in the community for her right now and here’s people you should speak to and never mind sending me like a chicken with my head cut off to 10 people. You need one navigator, one navigator and that person makes all the calls behind the scene. I was the navigator. (Caregiver 12)

#### Alzheimer’s society: Expanding role

Two caregivers spoke about how the role of the Alzheimer’s Society might be expanded to include additional services that target younger clientele.

The other thing that’s really lacking now and I’m not going to speak ill of the Alzheimer’s Society because they do, do good work but there are obviously opportunities for them to expand their programming too in my mind. They do a wonderful job with the First Link Program and getting information out about what to expect and different things that might occur. But a lot of that programming is geared towards older people. They need a program where I could have taken [name] by the hand and walked into the Alzheimer’s Society and sat down and had a coffee and listen to music or talk to other people. There was no program where I could bring her into the Alzheimer’s Society and play a game with staff or whatever, to give her stimulation, there was nothing. So I see that as a real gap in the Alzheimer’s Society. (Caregiver 12)

They need to start doing things differently and they need to start looking at who your clientele is. I can tell you three women under the age of 60 down on the same floor as my sister. So we need to start looking at what are the things that are unique in people that are younger dealing with Alzheimer’s. Most of them are in the workforce. So these are the things, some of the gaps that are missing when you’re dealing with younger people…so we need to start developing the programs around that. (Caregiver 12)

#### Increased visibility

Participants noted the need to increase visibility of the Alzheimer’s Society. Venues suggested included radio, television, posters in physicians’ offices, and to a lesser extent, social media.

I guess make it more visible, where is it in the community that we see the Alzheimer’s Society…where are these senior’s places where they can go to access support? Just increase visibility. They’re all hidden. I don’t know where they are. (Caregiver 2)

They [Alzheimer’s Society] need a big education awareness program about what is available, who it’s available for and the lack of reach out to families shocked me. It shocked me! Once you’re there, there are some good facilities. There are doctors doing the sessions. [Name] does a wonderful job on the session she does. There are social workers there. That part of it is fine, the First Link [program]. (Caregiver 12)

#### Preferential treatment

Caregivers felt that the older adult with dementia should not have to wait for extended periods in waiting rooms to see a healthcare provider, but rather should be bumped ahead of the cue to avoid problems. This preferential treatment included places like doctors’ offices and emergency departments.

I don’t know if they realize and I try to speak for mom so many times but I don’t know if they realize just how tough it is to take them to a place like that and have to sit in a waiting room and all the other patients and to patiently wait when she can’t wait for anything. At that point she wasn’t waiting for anything and it was a real struggle to keep her sitting in a chair and she openly spoke out, ‘I’m not staying here. Come on, we got to go, we got to go’. Like could someone in this office see that this is not the way it should be for seniors? You know if you’re in an office and the first thing they see is your MCP card so they know your age. So if there are 15 people there that are 40 years old well knock the 80-year old or 90-year old to first spot. (Caregiver 6)

One suggestion from a caregiver was that someone from community health conduct a home visit for assessment and planning of future care. This would alleviate the stress and burden for caregivers and the person with dementia having to leave the home to seek answers.

Or even if they found out through the Alzheimer’s Society that we have this number of families who are struggling with it. Then, even having someone to come in to do an in-house session with them to say, ‘Listen, this is what we’ve identified through whatever and this is how we suggest that you deal with it or handle this for a person with Alzheimer’s’. So the people don’t feel they have to go out into the community but the community is actually coming to them. (Caregiver 5b)

## Discussion

Our study indicates that barriers to the use of formal care services for those in the early stages of dementia include: lack of available services, especially in rural areas; lack of knowledge and misunderstanding about dementia and available services such as those provided by the Alzheimer’s Society; hesitation of the person with early-stage dementia and caregivers to seek services; and the health care system, which is overloaded and often unprepared to care for this population. Strategies for improving awareness of, and increasing access to formal services centered primarily on increasing public knowledge about dementia and Alzheimer’s disease, and increasing services. The Alzheimer’s Society was viewed as an important source of information and support for those in our study, yet some felt the role of the organization could be expanded upon. Furthermore, many participants did differentiate Alzheimer’s disease from dementia and were therefore, unaware that the Alzheimer’s Society could be a place where they could avail of information. A public awareness campaign that would include clarification of this misperception would be beneficial.

Health and social policy tends to encourage people with dementia to live at home for as long as possible, however, this can only occur if informal care is complemented and supplemented with appropriate formal care. It is therefore important for governments to understand the reasons behind the non-use of formal care services and the associated social and economic consequences of this non-use. This research contributed to one of the four foci of the NL Dementia Care Action Plan 2023–2026 (Government of Newfoundland and Labrador, 2023), namely, to improve the supports and services that people need to live well with dementia. The beginning of dementia marks a time when older adults and their families have an urgent need for information and medical support. Our findings are consistent with a recent systematic review (Ma, Zhang & Zheng, 2023) which determined that during or after the onset of symptoms or diagnosis of dementia or mild cognitive impairment, little information about recommendations or follow-up support is provided to participants, which is why they often express ‘not knowing what to do’ and feeling abandoned. To enable those with early-stage dementia and their families to make informed decisions about care, they need information about the possible illness trajectory, different care alternatives, and the consequences of these alternatives. This need for information and follow-up care that is straightforward and easy to access is of great importance.

The National dementia strategy for Canada recommends care planning for people with dementia. Care planning is important to improve outcomes for care recipients in health and social care by facilitating participation and shared decision-making, supporting self-management, behavior change, and coordinating treatments (Burt et al., 2014). Care planning should be a multidisciplinary process, and an action-focused and solution-oriented collaboration, including goal-setting with the person and the caregivers at the center of the care plan and their caregivers (Coulter et al., 2015). Despite this, the evidence for care planning is not compelling. A Cochrane review (Reilly et al., 2015) found that case management approaches in care for home-dwelling persons with dementia may lead to reduced institutionalization and decreased burden and depression among caregivers. However, the results were inconsistent throughout repeated measurements. Similarly, a systematic scoping review by Low et al. (2023) on care planning for community-dwelling older adults with dementia revealed that there is limited evidence to support that care planning alone improves outcomes for people with dementia and their caregivers. The review indicated that care planning processes and topics are influenced by whom is carrying out the care planning and that the health and social care context can impact the plan. This suggests that care planning can be very contextual, and that care plans often have different goals.

Being an active partner in a health care system that is easy to navigate was deemed important to the participants in our study. Also important was a health care system that was ‘understanding’ of the issues. This is consistent with other research. For example, Carey et al. (2023) reported that people living with dementia in the community have a positive perception of the care they received from health care providers. However, improvements could be made in how health providers speak directly to the person living with dementia when exploring how they would like to be involved in treatment decisions. Another study revealed that people living with dementia value being followed by community aged care services: feeling as though they were collaborating in determining what services they would receive; having their needs understood; being encouraged to interact with their service providers; being listened to and the verbal and non-verbal communication of service providers (Gill et al., 2011). Yet people with dementia often feel they are not included in decision-making about their own care (Donnelly et al., 2019). People with dementia and their caregivers have reported that they do not receive sufficient support and information around diagnosis (Francis & Hanna, 2020; Low et al., 2018) and find services fragmented and difficult to navigate (Martin et al., 2020). These issues partly arise from the lack of integration and coordination between health and social service providers (Low et al., 2018).

Dementia is a complex condition and meeting the diverse and changing needs of people living with dementia and their families requires an evidence-based, individualized and coordinated approach to service delivery that recognizes the rights of people living with dementia to be included in any decision-making. Although the primary purpose of our study was to increase understanding of how persons living at home with early-stage dementia and their caregivers/significant others currently perceive and interact with health and community-based services and service providers, our findings fully support all four foci of the Government of NL Dementia Care Action Plan. This three-year action plan outlines actions to improve the lives of individuals and families living with dementia. Implementation of actions in the plan will increase awareness of dementia and create safe and accepting communities where individuals living with dementia remain active and engaged. The plan also focuses on improving diagnosis, coordination of care, and enhancing supports and services for individuals and families living with dementia (Government of NL, 2023).

## Limitations

A major limitation of this study was that data collection occurred in only one area of NL, representing only one of four health authorities in the province. Although we interviewed participants in both rural and urban areas in eastern NL, health care disparities do exist across all areas of the province, with other areas having more remote and rural communities than eastern NL. The findings however, may be transferable to other areas of the province. A second limitation was that data were gathered during the COVID-19 pandemic. The pandemic disrupted services across the health care system and this disruption may have been particularly challenging for older adults with dementia. Although we were not focusing on this aspect of the experience, the experiences as described were within, and possibly tainted by, the context of the COVID-19 pandemic. A third limitation of this study were the small number of key informants we were able to recruit. Again, we attributed this to the COVID-19 pandemic, whereby health providers such as nurses were stretched to meet the exceeding demands of the healthcare crisis.

## Conclusion

This study presented the perceived barriers and facilitators to the access to and uptake of formal support services of the older adults with mild dementia and their caregivers in one region of NL, Canada. Older adults with early dementia, caregivers and key stakeholders were interviewed. Early recognition of dementia through education and publicity, enhanced public awareness, attention, and social inclusion with dementia-friendly neighborhoods and facilities need to be considered to achieve effective dementia-related services. Inclusion and recognition of the wishes of persons with dementia is key. The findings contributed to developing a multi-disciplinary integrated action plan for the local health response across the province for dementia services.
